# Xuesaitong Combined with Dexmedetomidine Improves Cerebral Ischemia-Reperfusion Injury in Rats by Activating Keap1/Nrf2 Signaling and Mitophagy in Hippocampal Tissue

**DOI:** 10.1155/2022/5126042

**Published:** 2022-12-07

**Authors:** Guo-Jie Han, Xiang-Zhen Min, Shuang-Shuang Ma, Chuan Ding, Xiu-Qin Wang

**Affiliations:** Department of Anesthesiology, Shandong Cancer Hospital and Institute Affiliated to Shandong First Medical University (Shandong Academy of Medical Science), Jinan, 250117 Shandong, China

## Abstract

Ischemic stroke is the most common type of cerebrovascular disease with high mortality and poor prognosis, and cerebral ischemia-reperfusion (CI/R) injury is the main murderer. Here, we attempted to explore the effects and mechanism of Xuesaitong (XST) combined with dexmedetomidine (Dex) on CI/R injury in rats. First, a rat model of CI/R injury was constructed via the middle cerebral artery occlusion (MCAO) method and treated with XST and Dex alone or in combination. Then, on the 5th and 10th days of treatment, the neurological impairment was assessed using the modified neurological severity scores (mNSS), the 8-arm radial maze test (8ARMT), novel object recognition test (NORT), and fear conditioning test (FCT). H&E staining was performed to observe the pathological changes of the hippocampus. ELISA and related kits were used to assess the monoamine neurotransmitters and antioxidant enzyme activities in the hippocampus. The ATP, mitochondrial membrane potential levels, and qRT-PCR of genes related to mitochondrial function were determined to assess mitochondrial functions in the hippocampus and western blot to determine Keap1/Nrf2 signaling pathway and mitophagy-related protein expression. The results showed that XST combined with Dex significantly reduced mNSS, improved spatial memory and learning deficits, and enhanced fear memory and cognitive memory ability in CI/R rats, which was superior to single-drug treatment. Moreover, XST combined with Dex treatment improved hippocampal histopathological damage; significantly increased the levels of monoamine neurotransmitters, neurotrophic factors, ATP, and mitochondrial membrane potential; and upregulated the activities of antioxidant enzymes and the expression of mitophagy-related proteins in the hippocampus of CI/R rats. XST combined with Dex treatment also activated the Keap1/Nrf2 signaling and upregulated the protein expression of downstream antioxidant enzymes HO-1 and NQ. Altogether, this study showed that a combination of XST and Dex could activate the Keap1/Nrf2 signaling and mitophagy to protect rats from CI/R-related neurological impairment.

## 1. Introduction

As a common type of cerebrovascular disease, ischemic stroke accounts for approximately 80% of all strokes and is one of the leading causes of death and acquired disability [1, 2]. Cerebral ischemia and hypoxia can induce ischemic stroke, and currently, returning the blood supply to the brain by surgery or medication is the primary treatment for ischemic stroke [3]. However, restoration of blood flow in brain tissues after ischemic stroke inevitably induces cerebral ischemia-reperfusion (CI/R) injury, thereby aggravating the condition [4]. CI/R injury can result in neurological deficits, learning and memory impairment, cerebral infarction, and cerebral edema in patients [5, 6]. It was reported that CI/R injury not only participates in the pathological processes of multiple factors and pathways but also correlates with mitochondrial dysfunction, neurotransmitter disorders, ion metabolism imbalance, oxidative stress, inflammation, and apoptosis [7]. Despite the knowledge of the pathogenesis and progression of CI/R injury, there are no effective therapeutic drugs and regimens to reduce CI/R injury in patients with ischemic stroke. Therefore, it is urgent to explore effective therapeutic agents and regimens to relieve CI/R injury in ischemic stroke patients.

Apart from the center of cellular energy production, the mitochondria are also involved in cellular physiological activities such as cell signaling, differentiation, proliferation, and apoptosis [8]. Mitochondria have also been shown to be involved in the pathology of CI/R injury. During CI/R, the mitochondria undergo a series of changes, such as Ca^2+^ overload, overproduction of reactive oxygen species (ROS), and opening of mitochondrial permeability transition pores [9, 10]. The above changes have profound effects on neurons and brain tissues. For instance, excessive ROS and Ca^2+^ overload can decrease mitochondrial membrane potential, increase membrane permeability, and release cytochrome c from mitochondria into the cytoplasm, thereby promoting neuronal apoptosis [9]. Excess ROS and damaged mitochondria are also substrates for mitophagy induction [10]. In addition to degrading and recycling damaged mitochondria, mitophagy also prevents cellular damage caused by mitochondrial DNA mutations [11]. Mitophagy is closely associated with CI/R injury. Some studies have reported that mitophagy plays a role in the central protection of neurons, glial cells, and endothelial cells by reducing ischemic injury [12, 13]. For instance, Mao et al. stated that ligustilide exerted neuroprotective functions by activating PINK1/Parkin-dependent mitophagy and ameliorating CI/R-induced hippocampal nerve injury [14].

Oxidative stress is a key factor in the pathogenesis of CI/R injury [15]. CI/R-induced mitochondrial damage can overproduce ROS, and excessive accumulation of ROS can lead to redox imbalance in the body and induce oxidative stress [15]. Keap1/Nrf2 is an important regulatory pathway for the endogenous redox system in the body. Normally, the binding of Keap1 to Nrf2 in the cytoplasm inhibits the activity of Nrf2. When ROS is excessive in the body, Nrf2 dissociates from Keap1 and translocates to the nucleus to activate the transcription of downstream antioxidant enzyme genes like haem oxygenase 1 (HO-1), NQO1, superoxide dismutase (SOD), catalase (CAT), and glutathione peroxidase (GPx) [16]. The Keap1/Nrf2 signaling pathway plays an important role in CI/R injury. Specifically, activation of the Keap1/Nrf2 signaling pathway and its downstream genes can ameliorate CI/R-induced neurological injury in rats [17].

Panax notoginseng (Burk.) F. H. Chen is a traditional Chinese herbal medicine that can activate blood, resolve stasis, alleviate swelling, and relieve pain [18]. Modern pharmacological studies have revealed the anti-inflammatory, antioxidant, hypolipidemic, and neuroprotective effects of Panax notoginseng. Panax notoginseng saponins (PNS) are the major active ingredients of Panax notoginseng, including notoginsenoside R1 and ginsenosides such as Rg1, Re, Rb1, and Rd [19]. Xuesaitong (XST), a traditional Chinese medicine preparation containing PNS, is mainly applied to clinically treat stroke-induced hemiparesis. Clinical studies have reported that XST improves neurological impairment in patients with cerebral infarction [20]. Additionally, XST can reduce neurological dysfunction and pathological damage in CI/R rats [21]. In contrast, dexmedetomidine (Dex) is an anesthesia-assisted sedative and a potent and highly selective *α*2 adrenoceptor agonist [22]. Some studies have reported the neuroprotective effects of Dex in animal models of brain injury. For instance, Zhao et al. reported that Dex could relieve CI/R injury in rats by activating *α*2-adrenergic receptors and blocking JNK phosphorylation and caspase-3 activation [22]. Sun et al. claimed that the neuroprotective effects of Dex were achieved by suppressing spinal cord inflammation and neuronal apoptosis and alleviating spinal cord CI/R injury [23]. However, the efficacy and mechanism of XST combined with Dex on CI/R injury are still unclear.

This present study is aimed at investigating the effects and mechanisms of the combination of XST and Dex on CI/R injury rats. The middle cerebral artery occlusion (MCAO) method was adopted to build the CI/R injury rat model, and multiple behavioral tests were performed to assess the effects of XST combined with Dex on the neurological function of CI/R rats. We discovered that XST combined with Dex treatment improved neurological impairment in CI/R rats and exhibited neuroprotective effects. Subsequently, the roles of XST combined with Dex in oxidative stress and mitochondrial function in hippocampal tissue of CI/R rats were investigated, followed by investigations of the mechanisms of the Keap1/Nrf2 signaling pathway and mitophagy. Altogether, this study showed that the combined regimen could be effective in treating reperfusion injury caused by ischemic stroke.

## 2. Materials and Methods

### 2.1. Animals

A total of 100 healthy specific pathogen free (SPF) level Sprague-Dawley (SD) rats (age: 6-8 weeks and weight: 180 g-200 g) were purchased from Hunan SJA Laboratory Animal Co., Ltd. After one week of adaptive feeding in the SPF environment, the rats were divided into a sham group, CI/R group, Dex group, XST group, and Dex+XST group, with 20 rats per group. The procedures for animal care and use were approved by the Ethics Committee of the Shandong Cancer Hospital and Institute Affiliated to Shandong First Medical University (No. 2022007005), and all applicable institutional and governmental regulations concerning the ethical use of animals were followed.

### 2.2. Establishment of CI/R Injury Model in Rats

CI/R injury rat model was constructed using the MCAO method [24]. Briefly, the surgery was performed in a sterile environment. First, the rats were anesthetized with isoflurane gas (5% induction and 2.5% maintenance), fixed in a supine position on the operating table, and the hairs from their neck were removed. After disinfecting with 75% ethanol, the skin of the neck and its subcutaneous tissue were incised along the anterior midline of the neck, and the subcutaneous tissue and fascia were bluntly dissected to expose the right common carotid artery (CCA), external carotid artery (ECA), and internal carotid artery (ICA). Next, the proximal parts of ECA and CCA were ligated, and ICA was clamped with an arterial clamp. A small opening was punctured by a needle at the distal end of CCA ligation to introduce embolization coils. Then, the ICA arterial clamp was released, and the direction and the position of the embolization coils were adjusted. Subsequently, the embolization coils were allowed to enter the ICA through CCA until they met a slight resistance, which was confirmed by the professional experimenters that ICA was occluded so that the middle cerebral artery was occluded. After 2 h, the embolization coils were removed to initiate the reperfusion process, and the neck tissue and skin were sutured.

### 2.3. Interventions in Grouping

**Figure 1:**
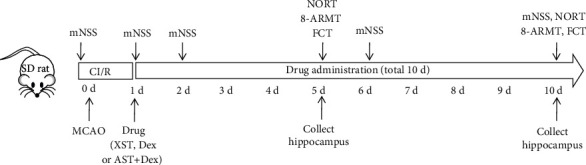
Experimental design of this study. For the sham group rats, only their blood vessels were exposed, and no embolization coils were introduced. Rats in the XST, Dex, and XST+Dex groups were treated medically every day after MCAO surgery for 10 days. The mNSS of rats in each group were measured before surgery and on days 1, 2, 6, and 10 of treatment. On the 5th and 10th days of treatment, behavioral tests were performed in each group of rats, followed by the collection of hippocampal tissues. On the 10th day of treatment, the hippocampal tissues were collected from rats in each group after completing behavioral tests; then, hematoxylin and eosin (H&E) staining was performed to observe histopathological changes in these tissues.

Dex hydrochloride injection was purchased from Jiangsu Hengrui Pharmaceuticals Co., Ltd. (H20090248) and XST from Hunan Fangsheng Pharmaceutical Co., Ltd. (Z20064307). XST tablets were dissolved in normal saline, followed by an intragastric administration. In the sham group, we only exposed the rats' carotid arteries but did not add the embolization coils. In the CI/R, Dex, XST, and Dex+XST groups, the rats underwent MCAO surgery. All rats received intervention treatment 24 h after surgery. Specifically, rats in the Dex group were intraperitoneally injected with Dex (50 *μ*g/kg) and gavaged with an equal volume of normal saline. Rats in the XST group were given XST via intragastric administration (40 mg/kg) and intraperitoneally injected with an equal volume of normal saline. Rats in the Dex+XST group received both intraperitoneal injection with Dex (50 *μ*g/kg) and intragastric administration with XST (40 mg/kg). Rats in the CI/R and sham groups were simultaneously injected intraperitoneally and gavaged with an equal volume of normal saline. All rats were treated once a day for 10 days, and rats that died during this period were excluded, and 10 rats in each group were randomly selected to continue the experiment. Behavioral tests were performed on days 5 and 10 of treatment. On the 10th day of treatment, after completing behavioral tests, the rats were sacrificed by deep anesthesia; then, the hippocampal tissues were collected from rats in each group; then, hematoxylin and eosin (H&E) staining was performed to observe histopathological changes in these tissues. The experimental design is shown in Figure 1.

### 2.4. Neurological Assessments

Before MCAO surgery and on days 1, 2, 6, and 10 of treatment, the degree of neurological impairment was assessed using a modified neurological severity score (mNSS) system by researchers blinded to the experimental design [25]. The score varied from 0 to 18, where 0 indicated normal neurological function and 18 indicated the most severe neurological impairment.

### 2.5. Eight-Arm Radial Maze Test

The spatial learning and memory abilities of the rats were evaluated with an 8-arm radial maze test (8-ARMT) [26]. Briefly, the 8-arm radial maze consisted of a central platform (diameter: 25 cm) and 8 extended arms (50 cm × 10 cm × 10 cm). A camera was placed 150 cm above the maze to track the movement trajectory of the rats. Besides, the central platform was connected with the arms through a controllable small door, and small boxes that could place food were set at the end of each arm. Before testing, the rats underwent adaptive training twice daily for 2 days, during which food was placed at the end of all eight arms of the maze, and the rats were free to explore for 10 min after entering the maze. In the formal test phase, the ends of the four arms were randomly selected for food placement, and the rats were allowed to freely explore the maze until all food at the ends of the four arms were eaten. The experiment was discontinued if the food was not finished after 10 min. After the test was completed, the maze was disinfected with alcohol and the residual odors were removed. Any-Maze™ software was applied to assess the rats' food exploration in the maze, based on the following: (1) working memory errors (WME): the number of reentries into arms where food was finished; (2) reference memory errors (RME): the number of entries into arms that did not contain food; and (3) total number of entries (TE): the total number of entries into the arms.

### 2.6. Novel Object Recognition Test (NORT)

The novel object recognition test (NORT) was performed to evaluate the rats' cognitive memory ability [27]. The test was conducted in a black open box (72 cm × 72 cm × 40 cm), and a camera was mounted above the box to record their movement trajectory. The experiment was divided into an adaptation phase, a training phase, and a testing phase. In the adaptation phase, the rats were placed in an open box and allowed to move freely for 10 min. In the training phase, the rats were put in an open box containing 2 identical objects for 10 min and allowed to freely explore the box. After 2 h, the formal testing started. Before testing, one of the objects in the box was replaced by a new object with a different shape, color, and material. The rats were allowed to freely explore the open boxes for 10 min; then, Any-Maze™ software was applied to analyze the exploration time of familiar objects (TF) and novel objects (TN) as well as the new object recognition index (RI) of rats. Notably, the alignment of the rats' noses to the object less than 2 cm away or directly touching the object was defined as exploring the object, which was calculated using the following formula: RI = TN/(TF + TN) × 100%.

### 2.7. Fear Conditioning Test (FCT)

The fear conditioning test (FCT) was adopted to assess the fear memory ability of rats [28]. Briefly, an electric shock generator and a sound generator were connected to a grating at the bottom of a fear conditioning chamber (Any-Maze, USA). Specifically, the rats were put in the fear conditioning chamber and given training. First, they were given cyclic noise stimulation (sound pressure level: 80 dB; frequency: 4 kHz; and duration/interval time: 30 s) and electrical shocks (current strength: 0.5 mA and duration time: 2 s). After 2 min, noise stimulation and electrical shocks were discontinued simultaneously. Then, the rats in the chamber were given context-related and tone-related tests 24 h later. In the context-related test, the rats moved in the chamber without noise and shock stimulation for 5 min. After 1 h, the tone-related test was conducted. After the rats had moved freely in the chamber for 3 min, they were given noise stimulation (duration/interval time: 30 s) for 5 cycles (total stimulation time: 5 min). The freezing behavior time of the rats in the stimulation process was recorded, and the Any-Maze™ software was utilized to analyze the freezing time percentage in the context-related and tone-related tests. Of note, except for respiration, indiscernible movements of all bones and vibrissa in rats were considered freezing behaviors.

### 2.8. Hematoxylin and Eosin (H&E) Staining

After rats were sacrificed, the brain tissues from five rats in each group were collected, and the hippocampal tissues were rapidly separated on the ice. After H&E staining, conducted according to the method described by Wang et al., the pathological changes in the rats' hippocampal tissues were observed [29]. Briefly, the tissues were fixed with 4% paraformaldehyde (Solarbio, China) for 48 h, followed by paraffin embedding to prepare 5 *μ*m of serial sections. Next, the sections were stained with H&E (Solarbio, China), following which they were observed and photographed under a microscope (Olympus, Japan).

### 2.9. Enzyme-Linked Immunosorbent Assay (ELISA)

After adding PBS buffer (Beyotime, China), the rats' hippocampal tissues (50 mg) were ground into tissue homogenate and centrifuged at 12000 rpm and 4°C for 30 min, and the supernatant was collected. The tissue homogenate was diluted to an appropriate concentration based on the instructions of dopamine (DA) kits (Enzyme-linked Biotechnology Co., Ltd., China), 5-hydroxytryptamine (5-HT) kits (Enzyme-linked Biotechnology Co., Ltd., China), norepinephrine (NE) kits (Enzyme-linked Biotechnology Co., Ltd., China), brain-derived neurotrophic factor (BDNF) kits (Enzyme-linked Biotechnology Co., Ltd., China), Trk B kits (Shenzhen Ziker Biological Technology Co., Ltd.), and neurotrophin-3 (NT-3) kits (SenBeiJia Biological Technology Co., Ltd., China). The reagents and samples were added to the ELISA plate in sequence, and after the reaction, the plate absorbance was detected at 450 nm. According to the standard curve, the levels of DA, 5-HT, NE, BDNF, Trk B, and NT-3 in the hippocampal tissue were calculated.

### 2.10. Assessment of Superoxide Dismutase (SOD), Catalase (CAT), and Glutathione Peroxidase (GPx) Activities

Similarly, the rats' hippocampal tissues were ground into a tissue homogenate after adding PBS buffer and centrifuged at 12000 rpm and 4°C for 30 min and the supernatant was collected. Related kits (Enzyme-linked Biotechnology Co., Ltd., China) were used to assess the activities of SOD, CAT, and GPx in the hippocampal tissues.

### 2.11. Detection of Adenosine 5′-Triphosphate (ATP)

PBS buffer was added to the rat's hippocampal tissues (50 mg), ground into tissue homogenate, and then centrifuged to collect the supernatant. Then, the ATP levels in the hippocampal tissues of the rats from each group were measured following the operating instructions of the ATP kit (enzyme-linked immunosorbent assay, China).

### 2.12. Mitochondrial Membrane Potential Detection

After sacrificing the rats, their hippocampal tissues were dissected on an ice disc and then rinsed with precooled Hank's solution (Solarbio, China). Subsequently, the pia mater and vessels were carefully removed under a dissecting microscope (Olympus, Japan), and the tissues were minced with iris scissors. Next, 0.125% pancreatin was added, and the tissues were placed in an incubator at 37°C for digestion. After 20 min, the digestion was discontinued by DMEM (Solarbio, China) containing 10% fetal bovine serum (FBS, Gibco, USA). The tissues were then sieved using a 200-mesh cell sieve and centrifuged at 1500 r/min for 5 min to collect the precipitated cells. Next, the cells were washed with Hank's solution, and the cell survival rate (not less than 90%) was detected using Trypan blue staining solution (Solarbio, China). Then, the cells were mixed with JC-I staining solution according to the operating instructions of the JC-I kit (Solarbio, China). Lastly, the fluorescence intensities at 490 nm and 530 nm were determined with a multifunctional microplate reader (TECAN, Switzerland).

### 2.13. qRT-PCR

Total RNA was extracted from the hippocampal tissues using the TRizol reagent (Sigma, USA), and the concentration and purity of RNA were detected using NanoDrop. The extracted RNA was reverse transcribed into cDNA according to the instructions of the reverse transcription kit (Thermo, USA). Then, the cDNA was utilized to synthesize target gene sequences on the Thermal Cycler Dice® Real-Time System following the SYBR GREEN kit (TaKaRa, Japan) instructions. GAPDH was used as the internal control, and the 2^-*ΔΔ*Ct^ method was adopted to calculate the relative expression levels of the target genes. The primer sequences applied in this study are shown in Table 1.

### 2.14. Western Blot

Total protein was extracted from the rats' hippocampal tissues using RIPA lysate (Solarbio, China), and the concentration of the extracted proteins was determined using BCA protein assay kits (Thermo, America). The protein was boiled with 5× SDS-PAGE protein loading buffer (Solarbio, China). Subsequently, 20 *μ*g of total protein was separated through sodium dodecyl sulfate-polyacrylamide gel electrophoresis (SDS-PAGE), transferred onto a polyvinylidene fluoride (PVDF) membrane, and blocked using 5% skimmed milk for 2 h. Next, the membrane was incubated with primary antibodies Keap-1 (cat. no. 60027-1-Ig), Nrf2 (cat. no. 16396-1-AP), HO-1 (cat. no. 66743-1-Ig), NQO1 (cat. no. 67240-1-Ig), PINK1 (cat. no. 23274-1-AP), Parkin (cat. no. 66674-1-Ig), LC3 (cat. no. 14600-1-AP), p62 (cat. no. 66184-1-Ig), and *β*-actin (cat. no. 66009-1-Ig), Proteintech, USA, overnight at 4°C. After washing with TBST thrice, the membrane was incubated with a secondary antibody (Abcam, USA) at ambient temperature for 2 h. Subsequently, enhanced chemiluminescence (ECL) reagents (PE0010, Solarbio, China) were evenly dripped onto the membrane, and a FluorChem HD2 imaging system was used to scan and photograph the blots. Grayscale value analysis was performed using ImageJ, and the relative expression levels of the target proteins were calculated with *β*-actin as the internal control.

### 2.15. Statistical Analysis

The SPSS v24.0 software was used for statistics and analysis and the GraphPad Prism v9 for plotting. All outcomes are presented as mean ± standard deviation (SD). Differences between the two groups were analyzed using independent *t*-test, and comparisons between multiple groups were assessed using a one-way analysis of variance. *p* < 0.05 was the criterion to indicate significant statistical difference.

## 3. Results

### 3.1. XST Combined with Dex Improves Neurological Impairment in Rats with CI/R

**Figure 2:**
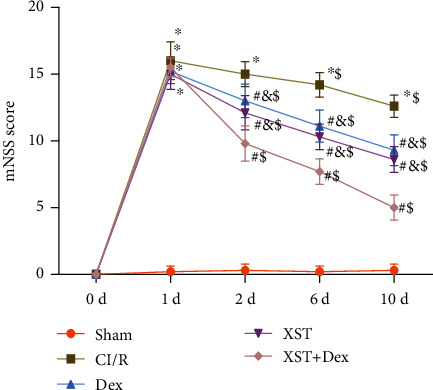
The combination of XST and Dex improves neurological impairment in rats with cerebral CI/R. Changes in each group before surgery and on the 1st, 2nd, 6th, and 10th days of treatment were assessed by the modified neurological severity score (mNSS) system (*n* = 10 per group). ^∗^*p* < 0.05 vs. the sham group at the same time points, ^#^*p* < 0.05 vs. the CI/R group at the same time points, ^&^*p* < 0.05 vs. the XST+Dex group at the same time point, and ^$^*p* < 0.05 vs. the same group of rats treated on the first day.

The mNSS system was used before and after treatment to explore the effects of XST combined with Dex treatment on the neurological function in CI/R rats. After MSCO surgery, the mNSS of rats in the CI/R group was significantly increased compared with that in the sham group (*p* < 0.05), indicating successful construction of the CI/R injury rat model of neurological impairment by MSCO surgery. On days 2, 6, and 10 after treatment, rats in the Dex, XST, and XST+Dex groups showed much lower mNSS than those in the CI/R group (*p* < 0.05); and compared with Dex and XST groups, the XST+Dex group presented significantly decreased mNSS (Figure 2). The above outcomes indicated that Dex combined with XST improved neurological impairment in CI/R rats, and the improvement of the combination treatment was better than that of the treatment alone. Further, the mNSS of rats in the XST+Dex group was decreased with the duration of treatment, suggesting that the improvement effect of XST+Dex on neurological impairment was increased with treatment duration.

### 3.2. Combination of XST and Dex Improves Spatial Learning and Memory Impairment and Enhances Cognitive Memory and Fear Memory Ability in CI/R Rats

**Figure 3:**
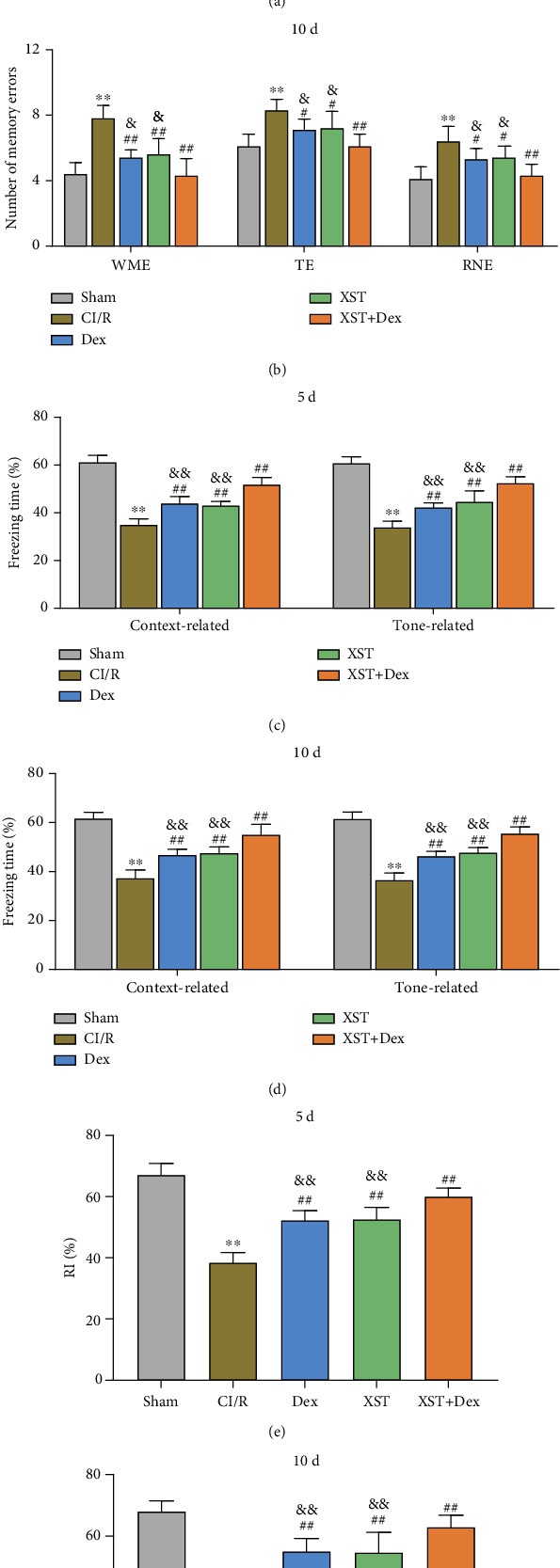
XST combined with Dex improves spatial learning and memory impairment and enhances cognitive memory and fear memory ability in CI/R rats. (a, b) Changes in working memory errors (WME), reference memory errors (RME), and total number of entries (TE) on the 5th (a) and 10th (b) days of treatment in each group of rats via 8-ARMT. (c, d) Fear conditioning test (FCT) to detect changes in the percentage of freezing time in context-related and tone-related tests in each group of rats on day 5 (c) and day 10 (d) after treatment. (e, f) Novel object recognition test (NORT) to check RI changes on the 5th (e) and 10th (f) days of treatment in each group of rats (*n* = 10 per group). ^∗∗^*p* < 0.01 vs. the sham; ^#^*p* < 0.05, ^##^*p* < 0.01 vs. the CI/R; and ^&^*p* < 0.05, ^&&^*p* < 0.01 vs. the XST+Dex.

CI/R-induced neurological impairment, characterized by learning and memory impairments and cognitive decline, is a common complication in ischemic stroke patients [5]. In this study, 8-ARMT was adopted to evaluate the role of XST combined with Dex in the spatial learning and memory abilities of CI/R rats. The evaluation results showed that the number of WME, TE, and RNE in the CI/R group was significantly increased compared with that in the sham group (*p* < 0.01), indicating that MCAO impaired spatial learning and memory abilities in rats. Moreover, on day 5 and day 10 of treatment, the Dex, XST, and XST+Dex groups exhibited a significant decrease in the number of WME, TE, and RNE compared with the CI/R group (*p* < 0.01, Figures 3(a) and 3(b)), suggesting that both single and combination therapies could improve spatial learning and memory abilities in CI/R rats. Additionally, on the 5th and 10th days of treatment, the number of WME, TE, and RNE in the XST+Dex group was lower than that in the Dex and XST groups (*p* < 0.05, Figures 3(a) and 3(b)), indicating better efficacy of the XST combined with Dex treatment on spatial learning and memory deficits in CI/R rats than that of the treatment alone.

Subsequently, the fear memory and cognitive memory abilities of rats in each group were assessed via FST and NORT experiments, respectively. According to the outcomes, compared with the sham group, CI/R rats demonstrated shorter freezing time in context-related and tone-related tests and much lower RI in the NORT test (*p* < 0.01), inferring that MCAO surgery significantly reduced fear memory and cognitive memory abilities in rats. Besides, Dex and XST, either alone or in combination, increased freezing time in context-related and tone-related tests and RI in the NORT test on the 5th and 10th days of treatment (*p* < 0.01). Furthermore, RI in the NORT test and freezing time in context-related and tone-related tests were significantly increased in the XST+Dex group compared with those in the Dex and XST groups (*p* < 0.01, Figures 3(c)–3(f)). The above outcomes suggested that combined treatment with XST and Dex improved cognitive memory and fear memory abilities in CI/R rats and was more effective than treatment with XST or Dex alone. Altogether, XST combined with Dex treatment improved spatial learning and memory deficits and enhanced cognitive memory and fear memory abilities in CI/R rats.

### 3.3. XST Combined with Dex Ameliorates Hippocampal Histopathological Damage in CI/R Rats

**Figure 4:**
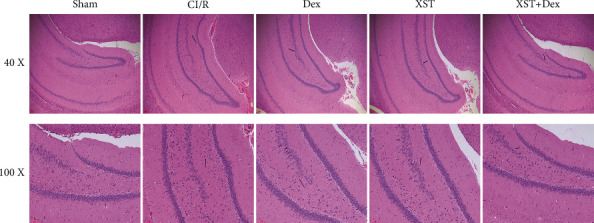
Combined treatment with XST and Dex alleviates pathological damage in the hippocampus of CI/R rats. Hematoxylin and eosin (H&E) staining was conducted to observe pathological changes of hippocampal tissue; 40x: scale bar = 100 *μ*m; 100x: scale bar = 10 *μ*m.

The hippocampus is involved in brain function activities, including learning, memory, cognition, and response control. Studies have revealed that cerebral ischemia can easily induce pathological damage to hippocampal tissues and impair neurological functions [30]. In this present study, H&E staining was performed to observe pathological changes in hippocampal tissues. Briefly, in the sham group, the cells had normal morphology, intact structure, and regular rank. As for the CI/R group, hippocampal tissue sections showed severe pathological damage, impaired vertebral cells, pyknotic and hyperchromatic neuronal nuclei, and the formation of vacuoles. Dex and XST, either treatment alone or in combination, improved the pathological damage of hippocampal tissues, and after treatment, the XST+Dex group rats had neatly arranged neurons and reduced cellular vacuoles in their hippocampal tissues (Figure 4). In sum, XST combined with Dex alleviated pathological damages in the hippocampus of CI/R rats.

### 3.4. Combined Treatment with XST and Dex Increases Monoamine Neurotransmitters and Neurotrophic Factor Levels in Hippocampal Tissue of CI/R Rats

**Figure 5:**
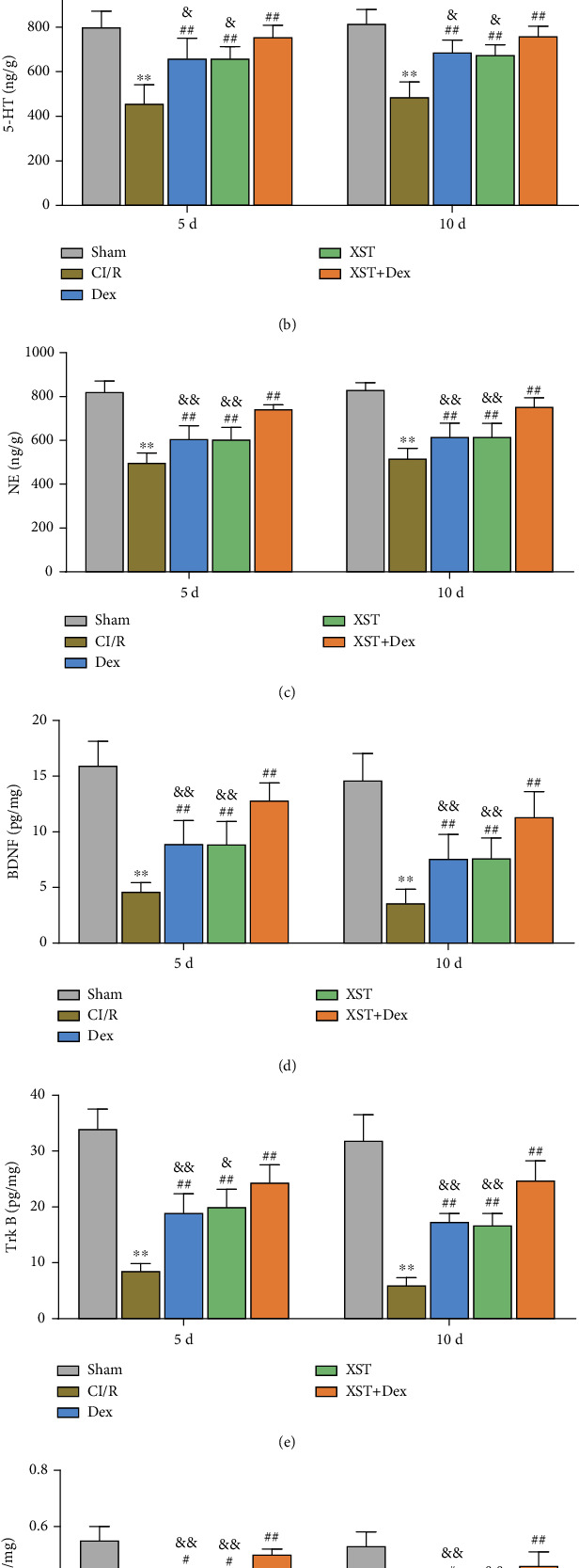
XST combined with Dex treatment increased the levels of monoamine neurotransmitters and neurotrophic factors in the hippocampal tissues of CI/R rats. (a–c) ELISA to detect changes in DA (a), 5-HT (b), and NE (c) levels in the hippocampus of rats on the 5th and 10th days of treatment. (d–f) ELISA to detect the changes in BDNF (d), TrkB (e), and NT-3 (f) levels in the hippocampus of rats after 10 days of treatment (*n* = 10 per group). ^∗∗^*p* < 0.01 vs. the sham; ^#^*p* < 0.05, ^##^*p* < 0.01 vs. the CI/R; and ^&^*p* < 0.05, ^&&^*p* < 0.01 vs. the XST+Dex. DA: dopamine; 5-HT: 5-hydroxytryptamine; NE: norepinephrine; BDNF: brain-derived neurotrophic factor.

Monoamine neurotransmitters and neurotrophic factors in the hippocampus are closely correlated with central nervous function. Specifically, monoamine neurotransmitters (DA, 5-HT, and NE) are involved in central nervous functions such as central cognition, memory, sensory, and sleep [31]. Neurotrophic factors (BDNF, Trk B, and NT-3) promote nerve growth and neurogenesis and exert neuroprotective effects in a rat model of CI/R injury [32]. It is unclear whether the neuroprotective effects of XST and Dex in CI/R-injured rats are associated with changes in monoamine neurotransmitters and neurotrophic factors in the hippocampal tissues. Therefore, ELISA was used in this study to determine the levels of monoamine neurotransmitters (DA, 5-HT, and NE) and neurotrophic factors (BDNF, Trk B, and NT-3) in the hippocampal tissues of CI/R rats after treatment with XST and Dex alone or in combination. The results showed that MCAO surgery significantly decreased the levels of DA, 5-HT, NE, BDNF, Trk B, and NT-3 in the rats' hippocampal tissues (*p* < 0.01). On the 5th and 10th days of treatment, XST and Dex alone or in combination increased the levels of DA, 5-HT, NE, BDNF, Trk B, and NT-3 in the hippocampal tissues of CI/R rats. Notably, the XST+Dex group presented significantly upregulated levels of monoamine neurotransmitters and neurotrophic factors in their hippocampal tissues compared with the XST or Dex groups (*p* < 0.05, Figures 5(a)–5(f)). Thus, Dex combined with XST treatment increased monoamine neurotransmitter and neurotrophic factor levels in the hippocampus of CI/R rats.

### 3.5. XST Combined with Dex Treatment Improves Oxidative Stress in Hippocampal Tissue of CI/R Rats

**Figure 6:**
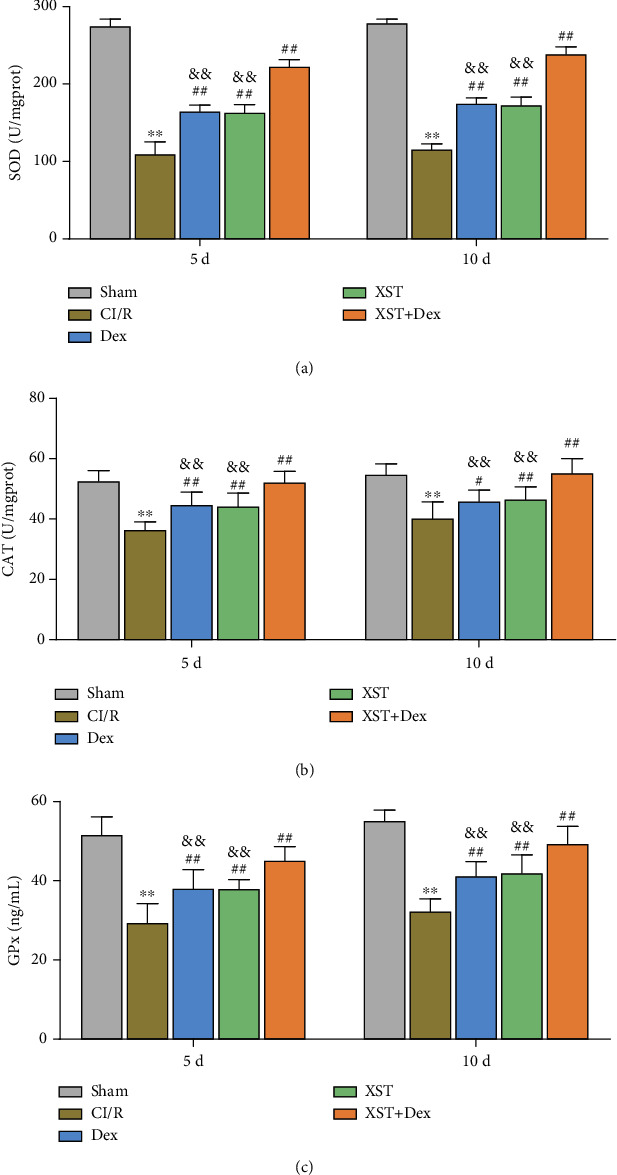
Improvements from XST combined with Dex treatment on oxidative stress on the hippocampal tissues of CI/R rats. (a–c) The levels of SOD (a), CAT (b), and GPx (c) in the hippocampus of rats in each group were measured by related kits on the 5th and 10th days of treatment (*n* = 10 per group). ^∗∗^*p* < 0.01 vs. the sham; ^#^*p* < 0.05, ^##^*p* < 0.01 vs. the CI/R; and ^&&^*p* < 0.01 vs. the XST+Dex. SOD: superoxide dismutase; CAT: catalase; GPx: glutathione peroxidase.

It is reported that oxidative stress is a key factor in CI/R injury [15]. To further explore whether the neurological improvement effect of XST combined with Dex treatment on CI/R rats was associated with oxidative stress, we examined the levels of antioxidant enzymes (SOD, CAT, and Gpx) in the hippocampal tissue of rats in each group. The results showed that the levels of SOD, CAT, and GPx in the hippocampus of the CI/R group rats were much lower than those in the sham group (*p* < 0.01), indicating increased oxidative stress levels in the hippocampus of CI/R rats. On the 5th and 10th days of treatment, the levels of SOD, CAT, and GPx in the XST, Dex, and XST+Dex groups were significantly increased compared with those in the CI/R group (*p* < 0.05), and the SOD, CAT, and GPx levels in the XST+Dex group were much higher than those in the XST and Dex groups (*p* < 0.01, Figures 6(a)–6(c)). The above findings indicated that XST combined with Dex could increase the antioxidant enzyme levels and ameliorate oxidative stress in the hippocampal tissues of CI/R rats.

### 3.6. Combined Treatment with XST and Dex Ameliorates Mitochondrial Dysfunction in Hippocampal Tissue of CI/R Rats

**Figure 7:**
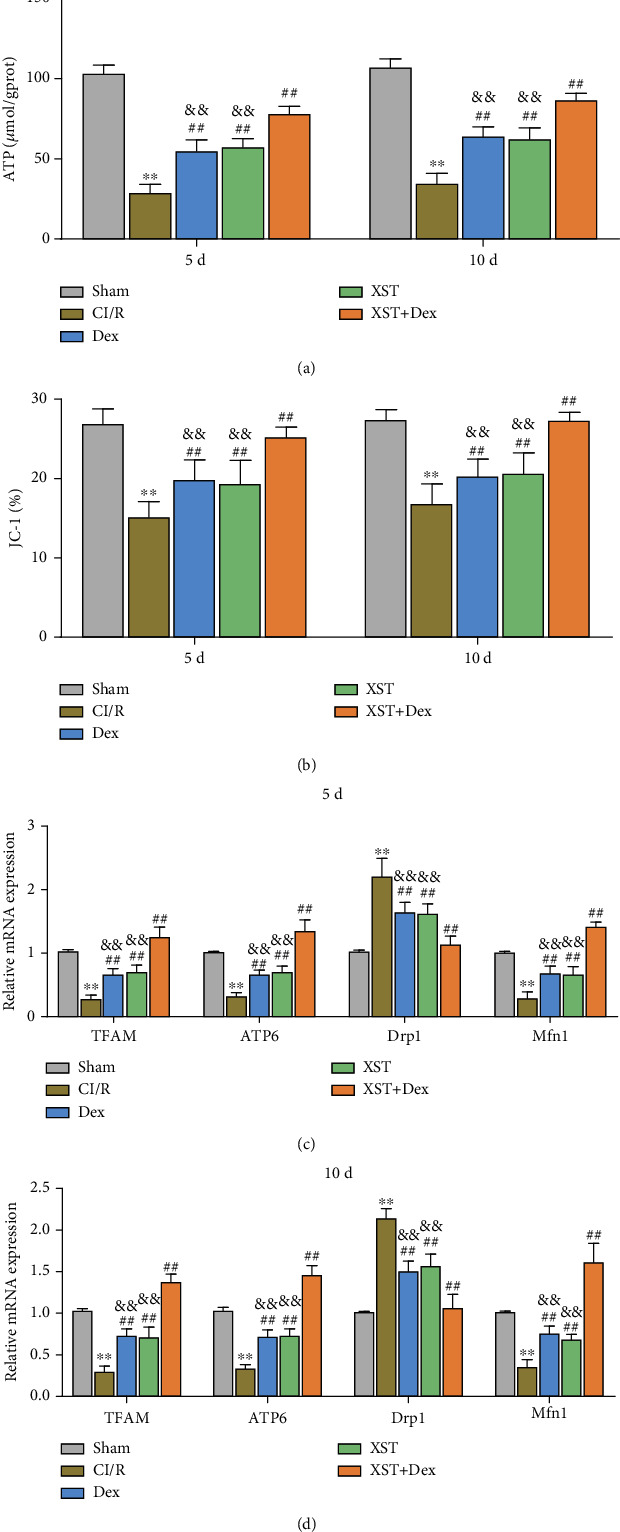
Improvements from XST combined with Dex treatment on mitochondrial dysfunction in the hippocampal tissues of CI/R rats. (a) Adenosine 5′-triphosphate (ATP) levels in the hippocampus of rats in each group were detected by related kits. (b) Mitochondrial membrane potential in the hippocampus of rats was detected using the JC-I kit. (c, d) qRT-PCR was performed to detect the mRNA expression levels of TFAM, ATP6, Drp1, and Mfn1 in the hippocampal tissues of rats in each group (*n* = 10 per group). ^∗∗^*p* < 0.01 vs. the sham; ^##^*p* < 0.01 vs. the CI/R; and ^&&^*p* < 0.01 vs. the XST+Dex.

Oxidative stress alters mitochondrial membrane potential and disrupts mitochondrial homeostasis. Alterations in mitochondrial function are associated with the pathological process of CI/R injury [9]. To explore the effect of XST combined with Dex treatment on mitochondrial function, we examined mitochondrial membrane potential and ATP levels in the hippocampal tissue of rats in each group. Briefly, compared with the sham group, mitochondrial membrane potential and ATP levels decreased notably in the CI/R group (*p* < 0.01). At day 5 and day 10 of treatment, mitochondrial membrane potential and ATP levels were significantly increased in the XST, Dex, and XST+Dex groups compared with the CI/R group. Compared with the XST and Dex groups, mitochondrial membrane potential and ATP levels in the XST+Dex group were much higher (*p* < 0.01, Figures 7(a) and 7(b)). All in all, XST combined with Dex increased mitochondrial membrane potential and ATP levels in the hippocampal tissues of CI/R rats.

Further, qRT-PCR was utilized to detect the mRNA expression levels of genes related to mitochondrial function (TFAM, ATP6, Mfn1, and Drp1) in the hippocampal tissues of rats in each group. The detection results displayed that, compared with the sham group, the mRNA expression levels of TFAM, ATP6, and Mfn1 were remarkably decreased, while the mRNA expression levels of Drp1 were significantly increased in the hippocampus of rats in the CI/R group (*p* < 0.01). On the 5th and 10th days of treatments with XST, Dex, or XST+Dex, the mRNA expression of TFAM, ATP6, and Mfn1 was upregulated, while that of Drp1 was downregulated in the hippocampus of CI/R rats. Compared with the XST and Dex groups, the XST+Dex group showed an increase in the mRNA expression of TFAM, ATP6, and Mfn1 (*p* < 0.01) and a reduction in the mRNA expression of Drp1 (*p* < 0.01, Figures 7(c) and 7(d)). Altogether, XST combined with Dex treatment improved mitochondrial dysfunction in the hippocampal tissues of CI/R rats, and the combination effects were better than single-drug treatment.

### 3.7. Combined Treatment with XST and Dex Activates Keap1/Nrf2 Signaling Pathway in Hippocampal Tissue of CI/R Rats

**Figure 8:**
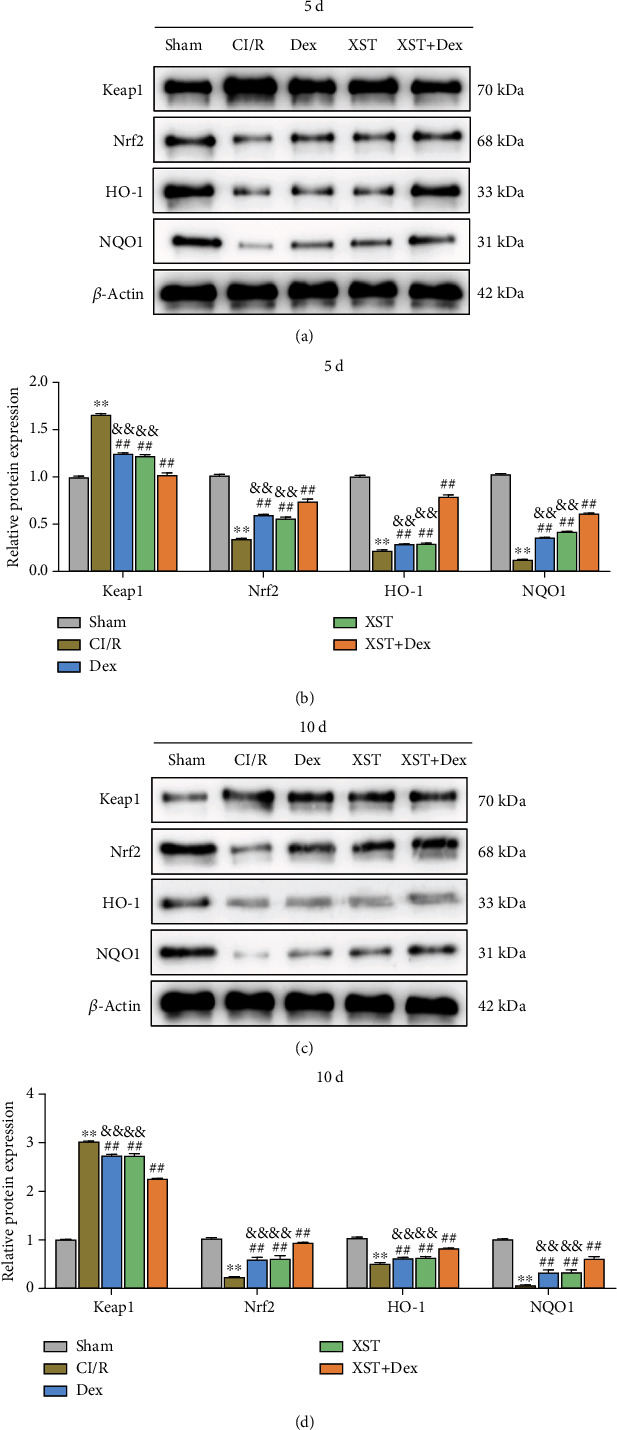
Combined treatment with XST and Dex activates the Keap1/Nrf2 signaling pathway in rats' hippocampal tissues. (a–d) Western blot to determine Keap1, Nrf2, HO-1, and NQO1 protein expression levels in the hippocampus of rats in each group on the 5th (a, b) and 10th (c, d) days of treatment (*n* = 3 per group). ^∗∗^*p* < 0.01 vs. the sham; ^##^*p* < 0.01 vs. the CI/R; and ^&&^*p* < 0.01 vs. the XST+Dex.

The Keap1/Nrf2 signaling is a key signaling pathway of the redox system in the body. The activation of Nrf2 and its downstream antioxidant enzyme genes (i.e., HO-1 and NQO1) can ameliorate CI/R-induced damage to the nervous system of rats [17]. To investigate the mechanism of XST combined with Dex treatment in improving CI/R injury in rats, western blot experiments were performed to detect the expression levels of Keap1/Nrf2 pathway-related proteins in the rats' hippocampal tissues. The outcomes showed that compared with the sham group, the protein expression of Keap1 was significantly increased (*p* < 0.01), while the protein expression of Nrf2, HO-1, and NQO1 was significantly decreased (*p* < 0.01) in the hippocampal tissues of the CI/R group. On day 5 and day 10 of treatment, the protein expression of Keap1 was significantly downregulated in the XST, Dex, and XST+Dex groups (*p* < 0.01), while the protein expression of Nrf2, HO-1, and NQO1 was significantly upregulated, compared with the CI/R group (*p* < 0.01). Moreover, compared with the XST and Dex groups, Keap1 protein expression was significantly downregulated (*p* < 0.01), while the protein expression of Nrf2, HO-1, and NQO1 was significantly upregulated in the hippocampus of rats in the XST+Dex group on the 5th and 10th days of treatment (*p* < 0.01, Figures 8(a) and 8(b)). These findings proved that XST combined with Dex treatment activated Keap1/Nrf2 pathway activity in the hippocampal tissue of CI/R rats.

### 3.8. Combination of XST and Dex Activates Mitophagy in Hippocampal Tissue of CI/R Rats

**Figure 9:**
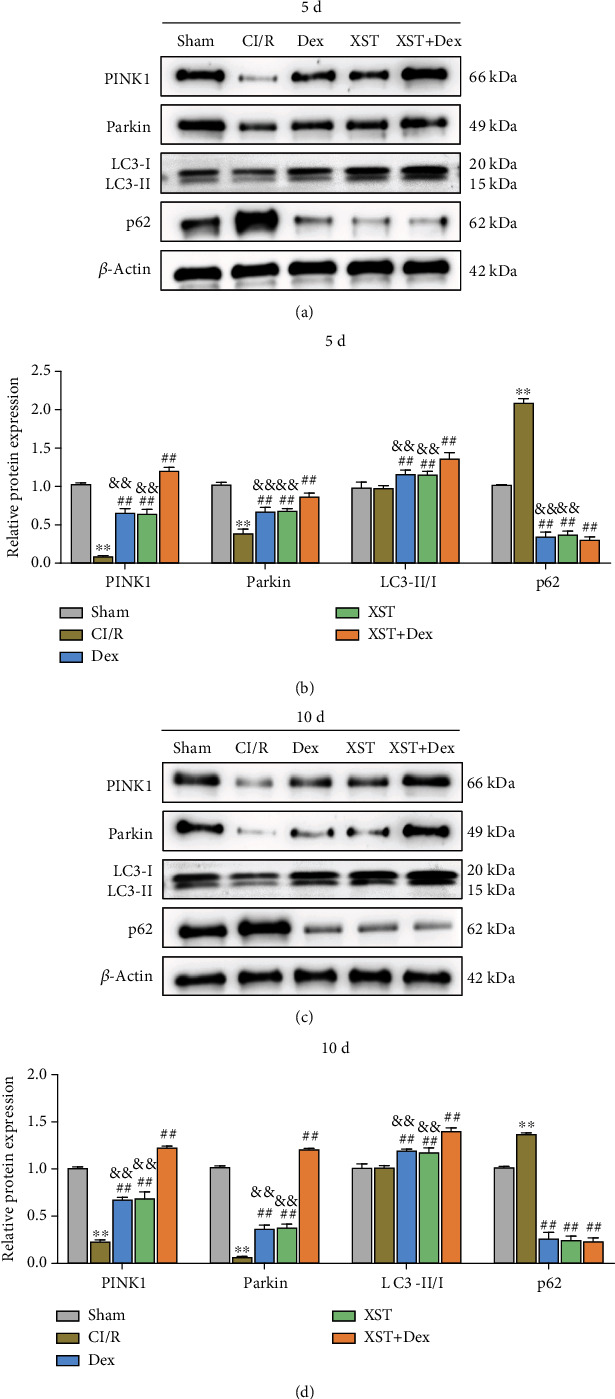
Combined treatment with XST and Dex activates mitophagy in the hippocampal tissues of CI/R rats. (a–d) Western blot to assess the protein expression levels of PINK1, Parkin, LC3-I, LC3-II, and p62 in the hippocampus of rats in each group on the 5th (a, b) and 10th (c, d) days of treatment (*n* = 3 per group). ^∗∗^*p* < 0.01 vs. the sham; ^##^*p* < 0.01 vs. the CI/R; and ^&&^*p* <0.01 vs. the XST+Dex.

Mitophagy is a physiological process whereby the body removes damaged mitochondria. PINK1/Parkin-dependent mitophagy can alleviate CI/R injury and exert neuroprotective effects [12]. To investigate whether the neuroprotective effects of combined treatment with XST and Dex on CI/R rats were associated with mitophagy, western blot was performed to assess protein expression changes related to mitophagy disorders in the rats' hippocampal tissues after XST and Dex treatment. Compared with the sham group, the results showed that PINK1 and Parkin protein expression levels were significantly decreased while the protein expression levels of p62 were increased in the CI/R group, suggesting that mitophagy was impaired in the hippocampal tissues of CI/R rats. On the 5th and 10th days of treatment, treatment with XST and Dex significantly increased the expression levels of PINK1 and Parkin and the value of LC3-I/LC3-II while the protein expression level of p62 was decreased in the hippocampal tissues of the CI/R rats. Furthermore, compared with the XST and Dex groups, the expression levels of PINK1, Parkin, and LC3-I/LC3-II were increased in the XST+Dex group (Figures 9(a)–9(d)). The above findings suggested that the combination of XST and Dex could improve mitophagy disorders in the hippocampal tissues of CI/R rats.

## 4. Discussion

In recent years, the incidence of ischemic stroke has been increasing worldwide. Restoring blood flow following an ischemic stroke can aggravate brain injury and trigger CI/R injury [4]. Currently, there are no effective clinical treatments and drugs to reduce CI/R injury. CI/R injury can induce neurological deficits, cerebral infarction, and cerebral edema [5, 6]. Some previous studies reported the functions of XST and Dex in protecting the nervous system and their roles in alleviating nerve injuries in CI/R rats [20–22]. In this study, MCAO surgery was found to increase mNSS in the rats. Further, compared with the sham group, the CI/R rats showed deficits in spatial memory and learning abilities and reductions in fear memory and cognitive memory abilities via 8-ARMT, NORT, and FCT. The above findings in our study are consistent with previous studies reporting on the effects of MCAO on neurological functions in rats [33]. Moreover, combined treatment with XST and Dex reduced mNSS in CI/R rats and improved behavioral disturbances and deficits of CI/R rats presenting in 8-ARMT, NORT, and FCT. Thus, XST combined with Dex demonstrated a synergistic effect in improving neurological impairment in CI/R rats and also exhibited better efficacy than treatment with XST or Dex alone.

The hippocampus is a key structure of the brain's limbic system, which participates in various functional activities in the brain. For example, the hippocampus plays an important role in learning, memory, and cognitive functions [29]. Generally, hippocampal tissues are susceptible to cerebral ischemic injury and are the main site of cerebral ischemia-induced histopathological changes [34]. In this study, the histopathological damage in the hippocampus of CI/R rats was severe and neural cells were vacuolated, while after XST combined with Dex treatment, the pathological damage in the hippocampus of CI/R rats was improved. Changes in the levels of monoamine neurotransmitters and neurotrophic factors are also important pathological changes induced by CI/R injury. Monoamine neurotransmitters play specific roles in central nervous functions such as cognition, memory, sleep, sensation, movement, and recovery of autonomic function [31]. Previous studies indicated CI/R could decrease DA, NA, and 5-HT levels in brain tissues [35, 36]. As for neurotrophic factors, they maintain neuroplasticity, affect brain functions, and regulate recovery after ischemic stroke [32]. BDNF, TrkB, and NT-3 have all shown neuroprotective effects in a rat model of CI/R injury [37]. Also, Gao et al. discovered that ginsenoside Rg2 could significantly increase DA, 5-HT, and NE levels in a rat model of posttraumatic stress disorder [38]. Similarly, recent studies have revealed that Dex can elevate the levels of monoamine neurotransmitters and neurotrophic factors [22, 39]. In this present study, we found that CI/R reduced DA, NA, 5-HT, BDNF, TrkB, and NT-3 levels in the rats' hippocampal tissues, which was consistent with that reported in previous studies [32, 35]. Additionally, XST combined with Dex treatment increased the levels of monoamine neurotransmitters and neurotrophic factors in the hippocampal tissues of IC/R rats. Furthermore, a synergy was observed between XST and Dex, whereby treatment with XST+Dex was superior to treatment with XST or Dex alone.

Mitochondrial functions are important indicators to determine the development of CI/R injury [9, 10]. Studies have pointed out that mitochondria are one of the important targets of ginsenosides, thereby contributing to the regulation of mitochondrial energy metabolism, oxidative stress, mitophagy, and the state of membrane channels [40]. Mitophagy is a normal mechanism for maintaining the physiological balance of cells. PINK1 is a serine/threonine protein kinase. In the normal state, PINK1 is transported from the cytoplasm to mitochondria and cleaved by mitochondrial proteases [14]. In CI/R injury, PINK1 is accumulated on mitochondrial membranes because decreased mitochondrial membrane potential makes PINK1 fail to translocate into mitochondria. Accumulation of PINK1 induces Parkin phosphorylation to activate Parkin. Activated Parkin binds its ubiquitin to OMM proteins, thereby inducing mitophagy. Also, some ubiquitinated proteins in the mitochondrial matrix bind to LC3 and anchor mitochondria to autophagosomes, thereby initiating mitophagy [14]. Dex can ameliorate CI/R injury by regulating the inhibition of mitochondrial calcium uniporter (MCU) and reducing excessive mitophagy [41]. In this report, MSCO decreased ATP levels, mitochondrial membrane potential, and the expression of mitochondrial function-related genes (TFAM, ATP6, Drp1, and Mfn1) in rats' hippocampal tissues and improved mitochondrial dysfunction. Besides, combination treatment with XST and Dex could activate PINK1/Parkin-dependent mitophagy in the hippocampus of CI/R rats. Previous reports have highlighted the potential role of XST and Dex in mitophagy [40, 41]. According to our study findings, XST combined with Dex was superior to XST or Dex alone in activating mitophagy and ameliorating mitochondrial dysfunction.

The Keap1/Nrf2 pathway is an important mechanism regulating oxidative stress in the body. Decreased Nrf2 activity may aggravate oxidative damage and inflammatory damage [42]. Recent studies reported a functional link between the Keap1/Nrf2 pathway and mitochondria [43]. Specifically, activation of Keap1/Nrf2 can enhance the activity of mitochondria, decrease the expression of NADPH oxidase system-related proteins, and inhibit mitochondrial oxidative stress through binding to *cis*-acting antioxidant response elements (ARE) [43]. Studies have shown that ginsenosides Rb1 and Rh3 can activate Keap1/Nrf2 signaling; upregulate the transcription and expression of HO1, NQO1, and GCLC; and exert antioxidant effects [44]. Liu et al. discovered that Dex significantly activated the Keap1/Nrf2/HO-1 pathway and relieved neuropathic pain in a murine model of sexual constrictive injury [45]. In our study, combined treatment with XST and Dex elevated antioxidant enzyme activity and improved oxidative stress status in the hippocampal tissues of CI/R rats. In addition, the combination of XST and Dex also activated Keap1/Nrf2 signaling and upregulated protein expression of downstream antioxidant enzymes HO-1 and NOQ1.

Despite the interesting discoveries reported in this study, there were some shortcomings. XST+Dex increased neurotransmitter levels in the hippocampus of CI/R rats, which may be associated with the synthesis and release of neurotransmitters in the central nervous circuit and central nervous system, which should be validated in further investigation. Additionally, the role of XST and Dex in improving neurological dysfunction caused by CI/R injury still needs to be demonstrated in larger clinical studies.

## 5. Conclusion

XST combined with Dex treatment improved neurological impairment, spatial memory, and cognitive dysfunction and enhanced cognitive memory and fear memory in CI/R-injured rats. Additionally, the combined treatment with XST and Dex activated the Keap1/Nrf2 signaling and mitophagy, ameliorated oxidative stress and mitochondrial dysfunction, and repaired mitochondrial damage in the hippocampus of CI/R rats.

## Figures and Tables

**Table 1:** Primer sequences of qRT-PCR.

Genes	Primer sequences (5′ to 3′)
TFAM	F: TCCTGTACTGAGCTGCC
	R: GAACATGTCTGCGTATCTC
ATP6	F: CCAGTGTTTAGACTATCTG
	R: GAACATGTCTGCGTATCTC
Drp1	F: GCGAACCTTAGAATCTGTGGACC
	R: CAGGCACAAATAAAGCAGGACGG
Mfn1	F: CCAGGTACAGATGTCACCACAG
	R: TTGGAGAGCCGCTCATTCACCT
GAPDH	F: GTCTCCTCTGACTTCAACAGCG
	R: ACCACCCTGTTGCTGTAGCCAA

## Data Availability

The data used to support the findings of this study are available from the corresponding author upon request.
